# Characterization of Wood Derived Hierarchical Cellulose Scaffolds for Multifunctional Applications

**DOI:** 10.3390/ma11040517

**Published:** 2018-03-28

**Authors:** Jana S. Segmehl, Vanessa Studer, Tobias Keplinger, Ingo Burgert

**Affiliations:** 1 Wood Materials Science, Institute for Building Materials (IfB), ETH Zürich, Stefano Franscini-Platz 3, 8093 Zürich, Switzerland; jana@segmehl-energie.de (J.S.S.); studerv@student.ethz.ch (V.S.); iburgert@ethz.ch (I.B.); 2 Applied Wood Materials Laboratory, EMPA–Swiss Federal Laboratories for Materials Science and Technology, 8600 Dübendorf, Switzerland

**Keywords:** wood-based cellulose scaffolds, delignification, acidic bleaching, soda pulping, Raman spectroscopy imaging, hygroscopic behaviour

## Abstract

Functional materials of high porosity and hierarchical structure, based on renewable building blocks, are highly demanded for material applications. In this regard, substantial progress has been made by functionalizing micro- and nano-sized cellulose followed by its reassembly via bottom-up approaches. However, bottom-up assembly processes are still limited in terms of upscaling and the utilization of these building blocks presupposes the disassembly of the plant feedstock inherit hierarchical cellulose scaffold. To maintain the three-dimensional structure, delignification processes from pulp and paper production were recently adapted for the treatment of bulk wood. Yet, a detailed chemical analysis and the determination of macroscopic swelling/shrinkage parameters for the scaffolds, necessary for a systematic design of cellulose scaffold based materials, are still missing. Here, acidic bleaching and soda pulping were used for producing cellulose scaffolds, for functional materials under retention of their inherent hierarchical structure. Spatially resolved chemical investigations on thin sections by Raman microscopy provided detailed information on the induced alterations at the cell wall level, revealing significant differences in dependence of the chemistry of the pre-treatment. An adaption to bulk wood samples proved the applicability of these treatments at larger scales and volumetric alterations at different atmospheric conditions indicated the effect of the altered porosity of the scaffolds on their hygroscopic behaviour.

## 1. Introduction

Multi-functional materials of light weight and mechanical stability are increasingly requested in our technologized world. Therefore, profiting from an improved understanding of structure-function relationships in natural materials, numerous bio-inspired solutions for the design of lightweight hierarchical synthetic materials were developed within the last years [1,2,3]. Furthermore, natural building blocks came into focus as structuring elements in materials research. In particular, nano-cellulosic materials have gained importance as reinforcing phase in novel composite systems due to significant advances in their preparation and functionalization [4,5].

Such cellulosic materials are mainly derived from plants in preparative processes, which are based on pulp production and usually comprise structural disintegration at different length scales [6]. This results in the loss of the three-dimensional multi-hierarchical organization, which is responsible for excellent properties, typical for natural materials and especially wood [7,8].

Therefore, it is desired to develop strategies to retain the native hierarchical structure found in plant tissues and profit from its structural organization and mechanical stability. Its high abundancy, facile workability, open porosity and mechanical integrity, makes wood an ideal sustainable resource to be used as structure providing scaffold in novel functional hybrid material systems [9,10,11,12,13,14].

A common limitation for functional materials based on native wood is that it is difficult to functionalize the very compact cell walls and therefore a strong functionalization often requires an insertion of modifying substances in the cell lumina. However, this goes along with an undesired increase in density. Hence, in order to reach an increased degree of functionalization under retention of the low density in the material, the additional phase has to be inserted into the cell wall or compound middle lamella. For an enhancement of the pore volume present in these structures chemical delignification processes adapted from pulp and paper industry are a promising approach. Rather recently, this concept was applied with subsequent delignification and functionalization for the development of transparent wood [15,16,17].

However, a spatially resolved chemical analysis of the cellulose scaffolds in dependence of the applied delignification treatment and the determination of physical properties (e.g., swelling properties) have not been performed yet. The chemical composition of the scaffolds on the cell wall level and the modified swelling behaviour strongly influence hydrophobicity and dynamic porosity and have in consequence a strong impact on efficient material integration.

In this work, two well established procedures for delignification have been examined regarding their potential as processes for the removal of lignin from the native cellulose scaffold present in wood. Based on an initial evaluation of environmental impact, toxicity, large scale applicability and process parameters (temperature, treatment duration), acidic bleaching and soda pulping were chosen. Previously applied in pulp and paper industry, acidic bleaching using a mixture of hydrogen peroxide and acetic acid and soda pulping with sodium hydroxide are known to be effective for the separation of lignocellulosic feedstocks into matrix (lignin and hemicelluloses) and fibre components [18,19,20]. In contrast to these treatments, which apply thorough comminution and intermixing during the process to ensure a homogeneous depletion, an adaption of the parameters is necessary to retain the structural integrity during scaffold preparation.

A detailed Raman spectroscopic study on thin sections was performed in order to understand how the different anatomical regions (cell wall, compound middle lamella) of the wood tissue are affected by the delignification processes. The separation into these two different anatomical regions is crucial to determine the ideal conditions for the scaffold preparation. While in the secondary cell wall a maximal lignin removal is envisaged to provide space for additional functionalizing material, the interconnecting layer between cells, the middle lamella and cell corner area, which consist mainly of lignin, should be preserved to retain the structural integrity of the scaffold. Alternatively, a selective removal of the compound middle lamella system followed by refilling with modifying agents of the latter could also be of high relevance. The hereby obtained modification would represent an interconnected system across the entire bulk wood sample.

Following this initial chemical investigation, the procedure was adapted to small bulk wood samples and optimized regarding suitable parameter combinations in terms of treatment temperature and duration. For the bulk samples, the homogeneity of the delignification over the whole sample volume, weight loss and dimensional changes upon humidity cycles were studied, as these parameters are crucial for the preparation of structurally stable macroscopic cellulose scaffolds.

## 2. Materials and Methods

### 2.1. Preparation of Wood Samples

Mature spruce wood with a similar growth ring pattern over the whole set of samples was used for a comparison of the different delignification treatments. For the initial chemical investigation with confocal Raman microscopy, cross-sections with areal dimensions of 5 × 5 mm^2^ and 20 µm thickness were prepared with a rotary microtome (RM 2255, Leica, Wetzlar, Germany). To study the properties of the three-dimensional scaffolds by determination of mass loss and hygroscopic dimensional changes, small cubes of the same material with size of 5 × 5 × 5 mm^3^ were prepared. For this purpose, long bars with a cross-section in transverse direction of 1 × 1 cm^2^ were cut with a circular saw and further sectioned into 5 mm thick disks, which were finally divided into 4 cubes using a razor blade.

### 2.2. Acidic Bleaching with H_2_O_2_/HAc

Hydrogen peroxide (H_2_O_2_, 30% analytical grade, Merck, Kenilworth Fort, NJ, USA) and acetic acid (99.8%, Sigma-Aldrich, Saint Louis, MO, USA) were mixed directly prior to the treatment at a volumetric ratio of 1:1. A total volume of 20 mL of the solution was used for the treatment of 10 thin sections. Heating to reaction temperature was conducted in an oil bath and the reaction solution was slightly stirred at 200 rpm. The treatment was conducted for 0.5, 2 and 4 h at 40 °C and 80 °C and for 0.25, 0.75, 1, 1.5 and 3 h at 60 °C. 

For 10 macroscopic wood cubes 50 mL of 1:1 H_2_O_2_/HAc solution was prepared. The treatment was conducted at 40 °C and 80 °C for 4, 6 and 8 h. 4 h for the thin sections respectively 8 h for the wood cubes were chosen as maximum reaction times as at longer reaction times sample disintegration was observed. To stop the treatment and remove access reactants, the samples were washed with Milli-Q water for a minimum of 7 days. The washing solution was regularly changed.

### 2.3. Soda Pulping with NaOH

A stock solution of sodium hydroxide (10 wt %) was prepared. 10 thin sections were introduced into a round bottom flask and 25 mL of stock solution were added. Heating to reaction temperature was conducted in an oil bath and the reaction solution was slightly stirred at 200 rpm. Durations of 0.5, 2 and 4 h at 40 °C, 60 °C and 80 °C were applied for the treatment of the thin sections. For the small macroscopic samples, 10 cubes were treated at a time under constant stirring in 50 mL of stock solution. The bulk material was heated to 40 °C and 80 °C for 4, 6 and 8 h. For comparability, the same treatment times were used for soda pulping. To stop the treatment and remove access reactants, the samples were washed with Milli-Q water for a minimum of 7 days. The washing solution was regularly changed.

### 2.4. Raman Analysis

Confocal Raman microscopy was conducted on the treated thin sections, sealed in MilliQ water between a glass slide and a cover slip (thickness = 0.17 mm). All measurements were recorded in backscattering configuration with an InVia Raman microscope (Renishaw, Wotton-under-Edge, UK) equipped with a motorized xyz stage. The excitation of Raman scattering was operated with a linearly polarized Nd-YAG laser (λ = 532 nm). A 100× oil immersion objective with numerical aperture (NA) 1.3 (Nikon, Tokyo, Japan) was used to maximize spatial resolution. The Raman light was detected by an air-cooled charge coupled device (CCD) camera behind a spectrometer (InVia) with a spectral resolution of about 1 cm^−1^. The mapping was recorded with an integration time of 0.2 s and a laser power of 23 mW in the spectral region between 300 and 1800 cm^−1^ and a step size of 300 nm.

The measurement setup as well as the initial data treatment, including cosmic ray removal and baseline correction, was done in Wire (version 4.1, Renishaw, UK). For the baseline correction, the inbuilt automatic intelligent background removal was used. After these initial steps, the data was further processed in the Matlab based software Cytospec (version 2.00.01). Regions of interest (ROI) from the secondary cell wall and the cell corner area of each measured mapping were extracted and averages were calculated. All cell wall average spectra were min/max normalized on the peak at 380 cm^−1^ in OPUS (version 7.2, Bruker, Karlsruhe, Germany). The obtained averages for one parameter set were averaged after careful examination to the final spectra presented.

### 2.5. Shrinkage and Swelling

For each treatment and each parameter set (temperature and treatment duration) 10 cubes were modified and analysed. The samples were cycled over four different climate conditions and the start point was the water saturation state directly after delignification. Subsequently, the dimensions of the samples were measured at 20 °C, 65% R.H. and 20 °C, 95% R.H., followed by a transfer to the wet state and a measurement point at 20 °C, 65% R.H. before final drying at 65 °C in a drying oven. For an adaption to the pre-set climate, 4 days of conditioning time was realized.

The resulting weight-loss, due to the performed delignification treatment, was recorded with a balance (PM4000, METTLER, Zürich, Switzerland). The wet states were excluded from this analysis, as the water film on the sample surfaces is critical to induce artefacts. Dimensional change was photographically recorded with a digital single lens reflex camera (PENTAX K-3 II, 90 mm objective, Pentax, Tokyo, Japan) in fixed distance to the sample. The images were analysed in ImageJ and the dimensional measures were retained for further calculations. The extracted data and the measured weight of the cubes were processed to calculate the averages, standard deviations and the density in Origin Pro 2016.

### 2.6. Light Microscopy

Longitudinal cuts and block faces in transverse direction were prepared through the centre of the modified specimen for investigations by optical microscopy in reflection. Microscopic investigations were conducted with an optical microscope (BX51, Olympus, Tokyo, Japan).

## 3. Results and Discussion

### 3.1. Comparative Chemical Investigation of Acidic Bleaching and Soda Pulping of Spruce Wood Cross Sections by Confocal Raman Spectroscopy

Norway spruce was selected as biological substrate for the preparation of the hierarchical wood derived scaffolds. The regular cellular arrangement renders it an appropriate model system for fundamental investigations at the cell and cell wall level. Further, the dominating volume fraction of tracheids provides a homogeneous tissue structure and excludes influences of for example, specialized liquid conducting pathways, such as vessels which are present in hardwoods [21].

In a first step, soda pulping and acidic bleaching were applied on thin sections using different reaction conditions and these were evaluated for chemical variations. The different parameter combinations applied for each treatment are provided in the Materials and Methods chapter.

**Figure 1:**
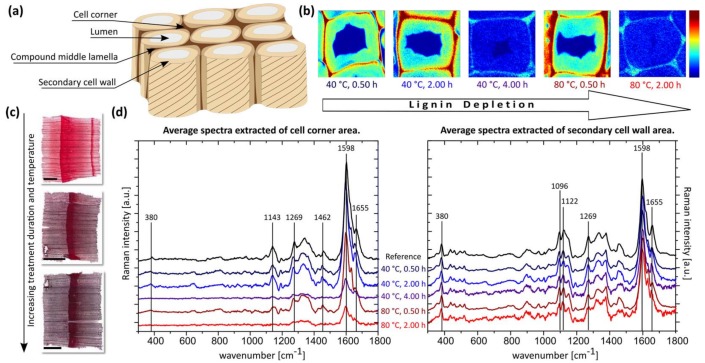
Characterization of soda pulped spruce wood cross sections. (**a**) Wood tissue model visualizing the main anatomical regions. (**b**) Raman images based on the integration of the main lignin band at 1598 cm^−1^ (all images are identically scaled). (**c**) Staining of spruce wood cross sections (scale bar = 1 mm). (**d**) Calculated average spectra, extracted from the secondary cell wall region and cell corner area (all cell wall spectra are normalized on the band at 380 cm^−1^).

**Figure 2:**
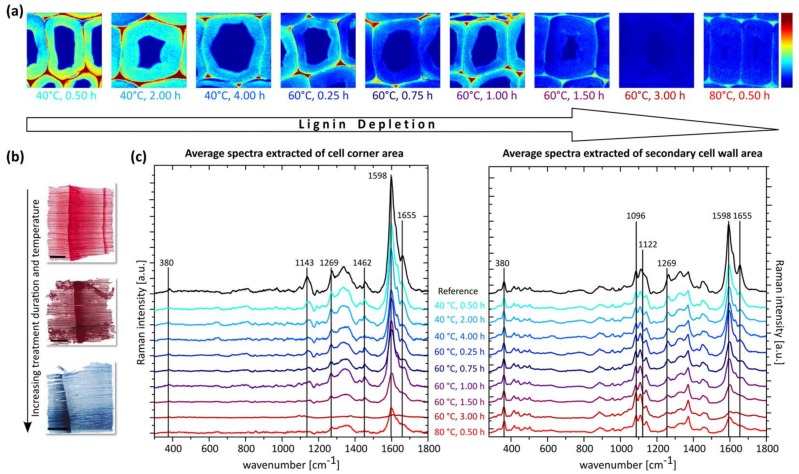
Characterization of acidic bleached spruce wood cross sections. (**a**) Raman images based on the integration of the main lignin band at 1598 cm^−1^ (all images are identically scaled). (**b**) Staining of spruce wood cross sections (scale bar = 1 mm). (**c**) Calculated average spectra, extracted from the secondary cell wall region and cell corner area (all cell wall spectra are normalized on the band at 380 cm^−1^).

To obtain a visual analysis of the induced alterations of the applied treatment on thin sections, staining with safranin red and methylene blue, colouring cellulose blue and lignified tissue red, was applied. The resulting coloration of the samples was homogeneous for all treatments and an indication for a reduction in lignin, visualized by a diminished red and enhanced blue portion, with increasing temperature and treatment duration. While the observed red discoloration, as a marker for a reduction in lignin was less pronounced for soda pulped sections (Figure 1c), it was close to complete after acidic bleaching (Figure 2b).

In soda pulping, in addition to lignin fragmentation, condensation reactions of residual lignin with fragments present in the structure are likely to happen. These reactions are competitive to fragmentation and counteract the lignin degradation through the formation of non-cleavable carbon-carbon bonds [22,23].

Subsequently, a detailed chemical analysis of the thin sections was performed by confocal Raman spectroscopy imaging. The analysis of the spectral data of each parameter set was separately performed for both, the secondary cell wall and cell corner region (Figure 1b,d and Figure 2a,c).

For the samples prepared by soda pulping no depletion of aromatic compounds in the secondary cell walls was observed. Even under harsh process conditions the peak intensity at 1598 cm^−1^, the main lignin vibration attributed to the aromatic ring stretching remained. This is supported by the unaltered band at 1655 cm^−1^, which is assigned to unsaturated C=C double bonds, a relative measure for crosslinking density of lignin [24,25,26,27].

Slight changes in the band at 1630 cm^−1^, assigned to aldehyde functionalities and a decrease of the band at 1269 cm^−1^, a marker band of the aryl-OH and aryl-OCH_3_ in guaiacyl units of lignin, point towards a reorganization on the molecular level. This results in the formation of non-cleavable carbon-carbon bonds due to re-condensation and densification of the lignin structure (Figure 1) [22,23].

The separated analysis of the cell corner region revealed a substantial degradation of this interconnecting region (Figure 1d). Although no significant reduction in the aromatic ring vibration was observed for short reaction times respectively low temperatures, for the sample treated at 80 °C/2 h and 40 °C/4 h a depletion in aromatic species was clearly present and confirmed by a similar decrease of the band intensity a 1269 cm^−1^, assigned to the guaiacyl (G) units in lignin. This enhanced effect of the alkaline delignification treatment on the cell corner area can be advantageous for the above-mentioned possibility of a selective compound middle lamella modification in order to achieve a homogeneous and continuous functionalization of the bulk wood material.

In contrast, for the acidic bleaching procedure the decrease of the main lignin vibration at 1598 cm^−1^, observed in the secondary cell wall, was strongly correlated with increasing temperature and treatment duration. Therefore, a gradual depletion of aromatic compounds is concluded (Figure 2c). The continuous removal of aromatic species is further confirmed by the constant reduction of the band at 1269 cm^−1^, a marker for the prevailing monomeric unit in softwood lignin and the decrease in intensity of the peak at 1655 cm^−1^, which is assigned to unsaturated C=C double bonds, a relative measure for lignin crosslinking density.

The simultaneous increase of the aldehyde-specific band at 1630 cm^−1^, indicates a displacement of the substituents at the C atom in the phenolic entities. The reversion of this spectral behaviour at temperatures above 60 °C and long treatment duration can be interpreted as an enhanced fragmentation of the phenolic macromolecule, respectively residual removal through leaching [22,23]. Based on these results, a continuous fragmentation of the lignin macromolecule into small aromatic entities and a subsequent leaching from the structure is assumed to be the prevailing mechanism in lignin degradation.

In general, for acidic bleaching, a similar behaviour in lignin degradation is observed for the secondary cell wall and cell corner areas (CC). Similar to the secondary cell wall, also for the CC area a reduction for the major bands assigned to the aromatic moieties and the crosslinking between the monolignols was present (Figure 2c). Nevertheless, even at high temperatures and treatment durations, the induced depletion of lignin (the decrease in the 1598 cm^−1^ band) was less pronounced. Therefore, a certain retention of the glue-like layer between the single cells, formed by the middle lamella and cell corner, can be maintained.

For both delignification processes, no disintegration of the treated sections was observed independent of the process conditions, which indicates that a sufficient structural integrity on the tissue level is preserved for both approaches. Moreover, for bands typically assigned to cellulose (380 cm^−1^, 1096 cm^−1^ and 1122 cm^−1^), no changes were detected irrespective of the chemistry of the applied procedure, which is beneficial for the application of the cellulose scaffolds in functional materials design [24,25].

### 3.2. Application of Acidic Bleaching and Soda Pulping as Delignification Procedures for the Preparation of Hierarchical Cellulose Scaffolds at a Small Macroscopic Scale

Following the detailed chemical analysis of thin sections, which addressed alterations on the molecular scale, investigations on the macroscopic samples with focus on physical variations were conducted. Essential for these investigations on a small macroscopic scale was an adaptation of the treatment in order to achieve a homogeneous delignification of the three-dimensional specimens. To compensate for longer diffusion pathways and temperature gradients across the specimens, the investigated treatment durations were significantly elongated. The respective process parameters for both chemical treatments are provided in the Materials and Methods chapter.

Visual observations, light microscopy and measurements of mass/volume changes were conducted. In order to meet the requirements of future materials applications, for example, maximize structural integrity and material integration and minimize leaching after functionalization, possible structural alterations due to swelling were examined.

**Figure 3:**
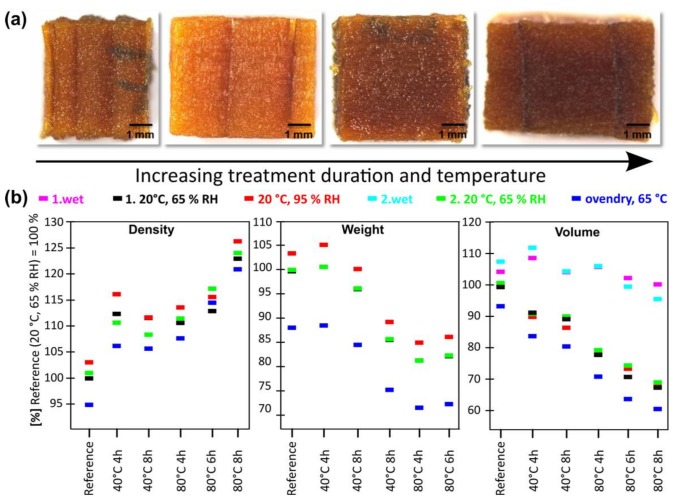
Characterization of soda pulped spruce wood cubes. (**a**) Light microscopy images of the soda pulped cubes of spruce wood. (**b**) Relative density variation, weight loss and volume changes for soda pulped samples treated under different conditions and monitored under the influence of changing climate conditions (colour coded).

**Figure 4:**
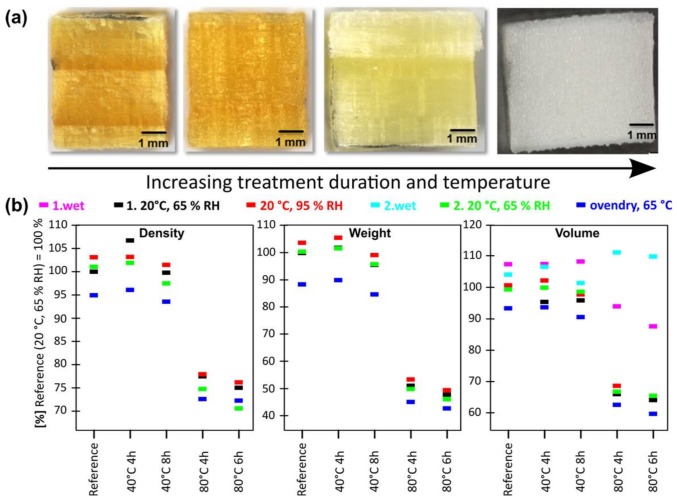
Characterization of acidic bleached spruce wood cubes. (**a**) Light microscopy images of the acidic bleached cubes of spruce wood. (**b**) Relative density variation, weight loss and volume changes for acidic bleached samples treated under different conditions and monitored under the influence of changing climate conditions (colour coded).

The initial visual investigation of the basic pulped cubes revealed a gradual darkening of the material in dependence of the applied process parameters (Figure 3a). This darkening of the soda pulped samples is a result of the initial formation of quinone intermediates, which are characterized by an intense dark-brownish colour [22,23]. In contrast, the acidic bleached cubes were characterized by a strong brightening due to the treatment (Figure 4a).

Cleavage of the cubes through their centre confirmed a homogeneous coloration for all samples which implies a homogenous alteration by both treatments, even for the three-dimensional scaffolds. In addition, the cubes maintained a sufficient mechanical and structural integrity which is crucial for further processing steps. To quantify the induced physical alterations, weight and volume were recorded in dependence of the applied treatment and process conditions and further monitored under cycling atmospheric conditions (Figure 3b, Figure 4b).

The climate cycle started in the initial wet state and included four different climate conditions, allowing to investigate the hygroscopic properties of the scaffold material. A comparison of the resulting weight of the treated cubes at oven dry conditions, revealed that the mass loss achieved by soda pulping did not exceed 15% (relating to the oven dried reference), even for the harshest process conditions (80 °C, 6 h). This relatively low amount of removed material confirms the previously obtained chemical data on thin sections, which exhibit only minor depletion of aromatic species within the cell wall.

The significantly higher weight loss of 50% relative to the initial mass monitored at oven dry conditions for the acidic bleached samples treated at 80 °C confirms the results obtained by Raman spectroscopy of thin sections. Considering an average lignin content in spruce wood of around 30%, it becomes evident that in addition to lignin also significant amounts of hemicelluloses and amorphous cellulose were removed, which is in accordance with previous studies [18].

A detailed investigation of the dimensional variations in dependence of the applied treatment and climate conditions was further conducted. For the soda pulped samples a gradual decrease of the volume in dependence of the process conditions could be observed (Figure 3b). Under the initial wet condition, the starting point of the climate cycle, the induced volumetric changes of the samples were negligible, indicating that the treatment does not result in a structural collapse prior to drying. However, already the first drying resulted in an enhanced shrinkage correlated with the applied treatment conditions and a large spread of the resulting dimensions. Rewetting of the material led to an almost complete recovery of the initial volume and a reversibility of the dimensional changes.

In contrast to the gradual decrease in volume observed for the soda pulped samples, the acidic bleached ones could be clearly divided into low and high temperature conditions (Figure 4b). Specimens obtained by acidic bleaching under low temperature conditions showed hygroscopic dimensional variations in the range of natural wood, while the dimensional stability of the high temperature treated samples was strongly decreased and a volume reduction up to 50% between initial wet state and oven dry state was observed.

The rewetting of the scaffolds in the fourth step of the climate cycle resulted in a full recovery of the initial wet state volume for the mildly treated samples, while the samples treated at elevated temperature did not fully retrieve their initial dimensions. Therefore, irreversible alterations in cell wall architecture have to be expected after strong matrix removal and subsequent drying. Independent of the severe impact of the applied treatment temperature, a similar volume was observed for all samples for the initial wet condition directly after delignification. This constant volume suggests that the matrix removal from the structural assembly takes place without a significant collapse of the inner cell wall structure.

Based on the obtained weight and volume data for each conditioning step, the density of the material was calculated. While for both applied procedures, a density reduction was recorded in the initial wet conditions, strong differences during drying and rewetting occurred. For the soda pulped material, a gradual densification of the material in the dry state was observed (Figure 3b), which makes an increase of porosity of the treated material rather unlikely. It can be assumed, that the reorganization of the matrix constituents in the treated material decreased dimensional stability and resulted in enhanced shrinkage upon water removal.

In acidic bleaching, a strong reduction in density was examined for the high temperature treatment, while material treated at low temperature showed a variability in density similar to unmodified wood (Figure 4b). This strong density reduction observed for acidic bleached samples, illustrates, that the induced weight loss is not linearly correlated with the observed shrinkage and an enhanced cell wall porosity can be assumed.

## 4. Conclusions

The presented high-resolution study on lignin removal by acidic bleaching and soda pulping showed that strong differences in the resulting material are obtained in dependence of the chemical protocols of the preparative treatment. By acidic bleaching an efficient depletion of aromatic constituents in the different anatomical regions was obtained. Nevertheless, even at a nearly complete removal of the lignin from the cell walls, a certain amount of the lignin remained within the compound middle lamella, which can still contribute to the structural integrity at the tissue level. In contrast, the soda pulped samples revealed no decrease of the aromatic content in the secondary cell wall region but a higher susceptibility to this treatment was observed in the cell corner/compound middle lamella area. Furthermore, the two procedures resulted in strong differences in swelling behaviour of the final scaffolds and showed distinct properties, which can be favourable for specific functionalizations. This includes the retained volume in the wet state after delignification treatment for the acidic bleached samples and the higher swelling capacity of the soda pulped samples.

**Figure 5:**
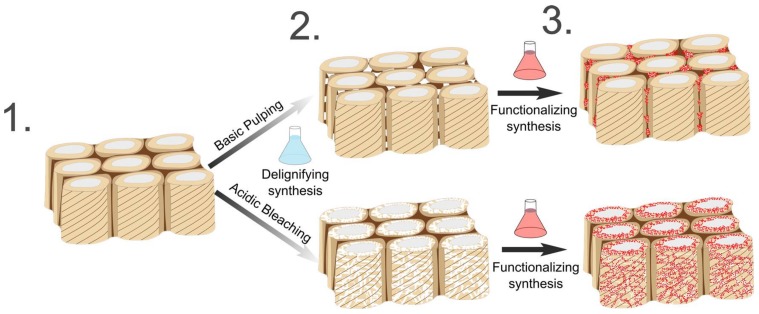
Preparation of wood-based hierarchical cellulose scaffolds for multi-functional applications. Native wood of variable dimensions (**1**) is immersed in the delignification bath to enhance porosity and cell wall accessibility on the cell wall level. The obtained wood-based cellulose scaffolds (**2**) can be functionalized via the integration of additional material into the induced porosity on the cell wall level. A preferential placement of the functional phase (**3**) can be guided in accordance to the anatomical response of the delignification treatment.

Hence, the delignification treatments could be selectively applied to achieve predestined cellulose scaffolds for targeted modifications, which preferentially allow for functionalization in between cells or in the cell walls, as illustrated in Figure 5. The presented top-down approach for the formation of hierarchical scaffolds represents a promising alternative to bottom-up strategies based on disassembly processes, which are energy-intensive and so far, limited in terms of up-scaling.
